# Joint Extraction of Entities and Relations Using Reinforcement Learning and Deep Learning

**DOI:** 10.1155/2017/7643065

**Published:** 2017-08-14

**Authors:** Yuntian Feng, Hongjun Zhang, Wenning Hao, Gang Chen

**Affiliations:** Institute of Command Information System, PLA University of Science and Technology, Nanjing, Jiangsu 210007, China

## Abstract

We use both reinforcement learning and deep learning to simultaneously extract entities and relations from unstructured texts. For reinforcement learning, we model the task as a two-step decision process. Deep learning is used to automatically capture the most important information from unstructured texts, which represent the state in the decision process. By designing the reward function per step, our proposed method can pass the information of entity extraction to relation extraction and obtain feedback in order to extract entities and relations simultaneously. Firstly, we use bidirectional LSTM to model the context information, which realizes preliminary entity extraction. On the basis of the extraction results, attention based method can represent the sentences that include target entity pair to generate the initial state in the decision process. Then we use Tree-LSTM to represent relation mentions to generate the transition state in the decision process. Finally, we employ *Q*-Learning algorithm to get control policy *π* in the two-step decision process. Experiments on ACE2005 demonstrate that our method attains better performance than the state-of-the-art method and gets a 2.4% increase in recall-score.

## 1. Introduction

Information extraction [1] is the task of automatically extracting entities, relations, and events from unstructured texts. Researchers usually do research on entity extraction, relation extraction, and event extraction as separated tasks, but in fact there are important dependencies among tasks. For instance, entity information can further help relation extraction, so relation extraction takes the results of entity extraction as input. If just using a pipelined approach to tackle the above problem, information from each task cannot interact and get any feedback. Therefore, we make a detailed study of joint extraction of entities and relations from unstructured texts, which can pass the information of entity extraction to relation extraction and obtain feedback in order to improve the performance of entity extraction and relation extraction simultaneously.

In recent years, more and more researchers have applied deep learning to entity extraction and relation extraction. Huang et al. [2] proposed a bidirectional LSTM with a CRF layer (BILSTM-CRF) for sequence tagging, which included part-of-speech tagging (POS), chunking, and named entity recognition (NER). Nguyen and Grishman [3] proposed to combine the traditional feature-based method and the convolutional and recurrent neural networks for relation extraction. Deep learning can automatically extract features of entities and relations between entities to replace the method of designing features manually. It reduces the dependence of external resources and achieves good performance.

But how to pass entity information to relation extraction and obtain feedback is the research focus to the task of joint extraction of entities and relations, which means that we need an effective combination of different deep learning methods. To tackle the problem, we use reinforcement learning to model the task as a two-step decision process. Because it is difficult to find some measures to directly represent the state from unstructured texts, we use some deep learning methods to extract the state in the process. Firstly, we regard entity extraction as a sequence tagging task and use bidirectional LSTM to capture the context information, which preliminarily realizes the tagging of entity state. On the basis of preliminary results, we use attention based method to represent the sentences that include target entity pair and generate the initial state *s*_1_ in the decision process, where the first decision is made. Then we use Tree-LSTM to capture the most important information of relation mentions and generate the transition state *s*_2_, where the second decision is made. The meaning of the two-step decision is as follows: the first decision is to judge if a sentence that includes target entity pair is a relation mention according to the preliminary results of entity extraction; the second decision is to classify the relation mention into a certain targeted type. By designing the reward function per step, entity information and relation information can interact. Finally, we use *Q*-Learning to get control policy *π* by maximizing cumulative rewards through a sequence of actions, which is essentially the mapping from state to action. In the training process of *Q*-Learning, all the parameters are jointly updated, which helps to realize the joint extraction of entities and relations. We conduct experiments on ACE2005 dataset and achieve better recall-score of both entity mentions and relation mentions than the state-of-the-art method. In the following, we define the task in Section 2 and present our method in Section 3. Then we detail an extensive evaluation in Section 4 and finally conclude in Section 5.

## 2. Task Definition

Our task is to extract all the entities and relations from unstructured texts simultaneously. In the section we randomly pick a sentence from ACE2005 dataset to analyze. The entity mentions and relation mention in the sentence are shown in Table 1, where Entity ID, Relation ID, and RefID are the identifications of mentions.

*Entity Extraction.* It can be taken as a sequence tagging task, which assigns a tag to each word *s*_*t*_ in the input sequence *S* = [*s*_1_, *s*_2_,…, *s*_*n*_]. The tag of a word means a combination of the entity type it belongs to and the boundary type it locates within. The boundary types are the Beginning, Inside, Last, Outside, and Unit of an entity (BILOU scheme). Table 1 shows two entity mentions in the sentence. The first entity mention is “third parties,” and its entity type is “ORG.” The second entity mention is “Entertainment,” and its entity type is “ORG.” ACE2005 dataset defines 7 coarse-grained entity types, which are “PER” (Person), “ORG” (Organization), “LOC” (Location), “GPE” (Geo-Political Entities), “FAC” (Facility), “VEH” (Vehicle), and “WEA” (Weapon). The types all have their own different subtypes.

*Relation Extraction.* It is to extract semantic relations of the targeted types between a pair of entities. Table 1 shows one relation mention in the sentence, of which the relation type is “ORG-AFF.” The first entity argument is “third parties,” and the second entity argument is “Entertainment.” The order of the arguments cannot be changed, which means the relation type is with direction. ACE2005 dataset defines 7 coarse-grained relation types between entities, which are “PHYS” (Physical), “PART-WHOLE” (Part-Whole), “PER-SOC” (Person-Social), “ORG-AFF” (Org-Affiliation), “ART” (Artifact), “GEN-AFF” (Gen-Affiliation), and “METONYMY” (Metonymy). Similarly, the types all have their own different subtypes.

*Joint Extraction.* It is to extract entities and relations in a sentence simultaneously. In the process of extraction, entity information and relation information can interact and get feedback information. Therefore, the joint extraction is more practical and different than separated entity extraction and separated relation extraction. We define and conduct research on the joint extraction task and present to use both reinforcement learning and deep learning for the task in the following section.

## 3. Our Method

The section combines three deep learning methods in the decision process of reinforcement learning for the joint extraction task. Firstly, we describe the two-step decision process; then we expound three deep learning methods used in this paper, that are bidirectional LSTM, attention mechanism, and Tree-LSTM; finally, we introduce *Q*-Learning algorithm that can get control policy *π*.

### 3.1. Reinforcement Learning

**Figure 1:**
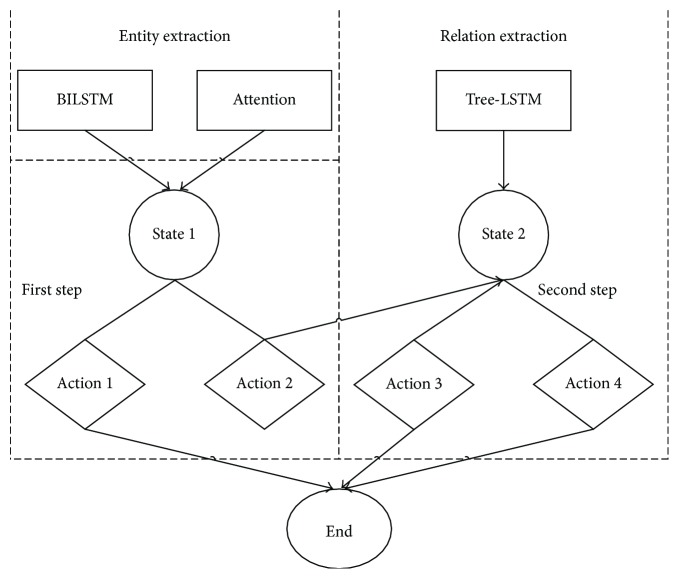
Two-step decision process.

In general, entity extraction is performed before relation extraction, and its results can also be taken as the input of relation extraction. Relation extraction is fundamentally divided into two stages: judge if a sentence that includes target entity pair is a relation mention; classify the relation mention into a targeted type. According to the thoughts, we model the joint extraction task as a two-step decision process by reinforcement learning. The two steps correspond to entity extraction and relation extraction roughly, and the specific flow is shown in Figure 1.

*Reinforcement Learning (RL).* It [4] is a commonly used framework for learning control policies by the agent, through interacting with its environment.

*State.* The internal state *S* in the environment consists of the initial state *s*_1_, the transition state *s*_2_, and the end state *s*_*e*_. Because it is difficult to find some appropriate measures to directly represent the state from unstructured texts, we use some deep learning methods to automatically extract features of texts, which can represent the state in the decision process. To be specific, we use bidirectional LSTM (Section 3.2) to realize preliminary entity extraction and use attention based method (Section 3.3) to generate the initial state *s*_1_ = Att(*X*; ***θ***_1_). In addition, we use Tree-LSTM (Section 3.4) to generate the transition state *s*_2_ = Tree(*X*; ***θ***_2_). The action taken at *s*_2_ realizes preliminary relation extraction. *X* is the features of the input sentence; ***θ***_1_ and ***θ***_2_ are parameters in the above models.

*Action.* There are a set of predefined actions *A* in the environment: Action 1 *a*_1_, Action 2 *a*_2_, Action 3 *a*_3_, Action 4 *a*_4_, and so forth. The first decision judges to take *a*_1_ or *a*_2_. *a*_1_ is to judge that a sentence that includes target entity pair is not a relation mention, and *a*_2_ is to judge that a sentence that includes target entity pair is a relation mention. The second decision judges to take *a*_3_ or *a*_4_…. *a*_3_ is to classify the relation mention into a targeted type, and *a*_4_ is to classify the relation mention into another targeted type. *R* = *r*_1_, *r*_2_, *r*_3_, *r*_4_,… denotes the reward obtained for each action. The agent takes an action *a* in state *s* and receives a reward *r* from the environment. (*s*_1_, *a*_1_, *r*_1_, *s*_*e*_), (*s*_1_, *a*_2_, *r*_2_, *s*_2_), (*s*_2_, *a*_3_, *r*_3_, *s*_*e*_), and (*s*_2_, *a*_4_, *r*_4_, *s*_*e*_) denote the transitions of the decision process.

*Transition and Reward Function.* A state transition tuple (*s*_1_, *a*_1_, *r*_1_, *s*_*e*_) means that the agent takes *a*_1_ at *s*_1_ and then transits to *s*_*e*_. If the judgement of *a*_1_ is right, then the agent receives a reward *r*_1_ = 10; if the judgement of *a*_1_ is wrong, then set *r*_1_ = −20 to punish the wrong judgement of the first decision. A state transition tuple (*s*_1_, *a*_2_, *r*_2_, *s*_2_) means that the agent takes *a*_2_ at *s*_1_, then transits to *s*_2_, and receives a reward *r*_2_ = 5. A state transition tuple (*s*_2_, *a*_3_, *r*_3_, *s*_*e*_) means that the agent takes *a*_3_ at *s*_2_ and then transits to *s*_*e*_. If the judgement of *a*_3_ is right, then the agent receives a reward *r*_3_ = 10; if the judgement on type is wrong, then set *r*_3_ = −10 to punish the wrong judgement of the second decision; if it is not a relation mention, then set *r*_3_ = −20. The meaning of other state transition tuple (*s*_2_, *a*_4_, *r*_4_, *s*_*e*_) and the definition of its reward function are similar to those of (*s*_2_, *a*_3_, *r*_3_, *s*_*e*_).

### 3.2. BILSTM

**Figure 2:**
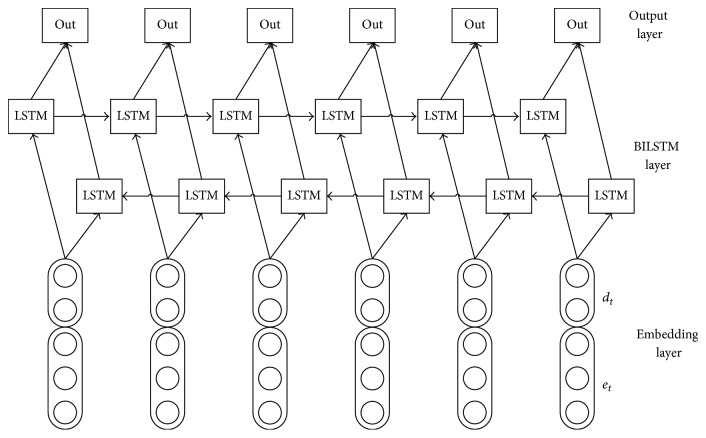
Basic structure of BILSTM.

Long Short-Term Memory (LSTM) [5] is a variant of recurrent neural networks (RNN) designed to cope with the gradient vanishing problem, and LSTM is very useful to find and exploit long range dependencies in the data. Now lots of LSTM variants have been proposed and applied to natural language processing tasks, such as sentiment analysis, relation classification, and question answering system. We use bidirectional LSTM (BILSTM) to model word sequence, which can efficiently make use of past features and future features. BILSTM finds the right representation of each word and assigns a tag of entity state to each word in the input sequence to realize preliminary entity extraction. BILSTM mainly consists of three representation layers: embedding layer, BILSTM layer, and output layer.  Figure 2 gives the basic structure of the BILSTM.

#### 3.2.1. Embedding Layer

The embedding layer converts discrete features of each word into continuous features as input of the BILSTM layer. We do forward and backward for input sentence, so we need a special treatment at the beginning and the end of the sequence.

Part-of-speech feature can further help entity extraction, so we only use word embedding *e*_*t*_ and part-of-speech embedding *d*_*t*_ to represent each word *w*_*t*_ in the input sentence, which replace the method of designing features manually. After passing through the lookup table, the lowercased word is mapped to its corresponding embedding. For word feature, the lookup table is initialized by the publicly available word embeddings. For part-of-speech feature, the lookup table is randomly initialized with values drawn from a uniform distribution. The word embeddings and the part-of-speech embeddings are allowed to be modified during training.

We concatenate the word embedding *e*_*t*_ and the part-of-speech embedding *d*_*t*_ of each word *w*_*t*_ to generate input feature vector *x*_*t*_ = [*e*_*t*_, *d*_*t*_]. The matrix *X* = [*x*_1_, *x*_2_,…, *x*_*n*_] represents the features of the whole sentence, and is passed to the BILSTM layer, where n is the length of the input sentence.

#### 3.2.2. BILSTM Layer

Basically, each LSTM unit in the BILSTM layer is composed of three multiplicative gates: an input gate *i*_*t*_, a forget gate *f*_*t*_, and an output gate *o*_*t*_. The gates can control the proportions of information to forget and to pass on to the next time step. In addition, there is a memory cell *c*_*t*_ in each LSTM unit, which can keep the previous state and memorize the features of the current input word. Therefore, the data sources of each LSTM unit are as follows: the feature vector *x*_*t*_ = [*e*_*t*_, *d*_*t*_] at time *t*, the hidden state vector *h*_*t*−1_ before time *t* or *h*_*t*+1_ after time *t*, and the cell vector *c*_*t*−1_. The forward passes are implemented as follows:

(1) it=σWxixt+Whiht−1+Wcict−1+bi,ft=σWxfxt+Whfht−1+Wcfct−1+bf,gt=tanh⁡Wxcxt+Whcht−1+Wccct−1+bc,ct=itgt+ftct−1,ot=σWxoxt+Whoht−1+Wcoct+bo,ht=ottanh⁡ct,

where *W* are weight matrices, *b* are bias vectors, and their subscripts have the meaning as the name suggests. *σ* denotes the logistic function.

The backward passes over time are carried out in a similar way to forward passes. The hidden state vectors of two directions *h*_*t*_ and *h*_*t*_′ are simultaneously computed at time *t* in the BILSTM layer, so we can efficiently make use of past features and future features for a specific time frame.

#### 3.2.3. Output Layer

We treat entity extraction as a sequence labeling task. By assigning an entity tag to each word, we realize preliminary entity extraction on top of the BILSTM layer. At time *t*, we pass the hidden state vectors of two directions *h*_*t*_ and *h*_*t*_′ to a softmax layer.

(2) $$\begin{array}{c} y_{t}=\text{softmax}\left(\;W_{hy}h_{t}+W_{h^{\prime }y}h_{t}^{\prime }+b_{y}\;\right). \end{array}$$

Here, *W* are weight matrices and *b* is bias vector.

#### 3.2.4. Objective Function

We employ the Viterbi algorithm to inference the tag sequence *T* = [*t*_1_, *t*_2_,…, *t*_*n*_] for a given input sentence *W* = [*w*_1_, *w*_2_,…, *w*_*n*_]. To model the tag dependency, we use the transition score **A**_*ij*_ for measuring the probability of the transformation from tag *i* to tag *j*. Thus, the sentence-level score can be formulated as follows:

(3) $$\begin{array}{c} s\left(\;W,T,\theta_{0}\;\right)=\overset{n}{\underset{i=1}{\sum }}\left(\;A_{t_{i-1}t_{i}}+y_{i}\left(\;t_{i}\;\right)\;\right). \end{array}$$

Here, *y*_*i*_(*t*_*i*_) is the score for choosing tag *t*_*i*_ for the *i*th word in the input sentence. ***θ***_0_ is the parameter set of BILSTM.

For a given training instance (*W*_*i*_, *T*_*i*_), *W*_*i*_ is a given sentence and the correct tag sequence for *W*_*i*_ is *T*_*i*_. We search for the tag sequence with the highest score:

(4) $$\begin{array}{c} T^{*}=\underset{\hat{T}}{\mathrm{argmax}}\;s\left(\;W_{i},\hat{T},\theta_{0}\;\right). \end{array}$$

Here, $\hat{T}$ is a predicted tag sequence.

The regularized objective function for *m* training instances is the loss function *J*(***θ***_0_) including a *l*_2_-norm term:

(5) Jθ0=1m∑i=1mliθ0+λ2θ022,liθ0=max⁡0,sWi,T^i,θ0+ΔTi,T^i−sWi,Ti,θ0.

Here, $\Delta \left(T_{i},{\hat{T}}_{i}\right)$ is a structured margin loss for predicted tag sequence $\hat{T}$. *λ* is an *L*2 regularization hyperparameter.

To minimize *J*(***θ***_0_), we use a generalization of gradient descent called subgradient method [6] which computes a gradient-like direction.

### 3.3. Attention Mechanism

Recently, attention mechanisms have successfully been applied to machine translation [7], text summarization [8], text comprehension [9], syntactic constituency parsing [10], relation classification [11], and text classification [12]. Inspired by those studies, we introduce attention based method to compute the hidden state vectors *h*_*t*_ and *h*_*t*_′ in the BILSTM layer and generate the initial state *s*_1_ in the decision process. The method can obtain the information of entity extraction and represent the sentences that include target entity pair. After the first decision on *s*_1_, we realize preliminary entity extraction and get ready to perform relation extraction. In essence, attention based method can pass entity information to relation extraction and obtain feedback information of relation extraction by jointly updating all the parameters. Attention based method better integrates entity extraction and relation extraction.

**Figure 3:**
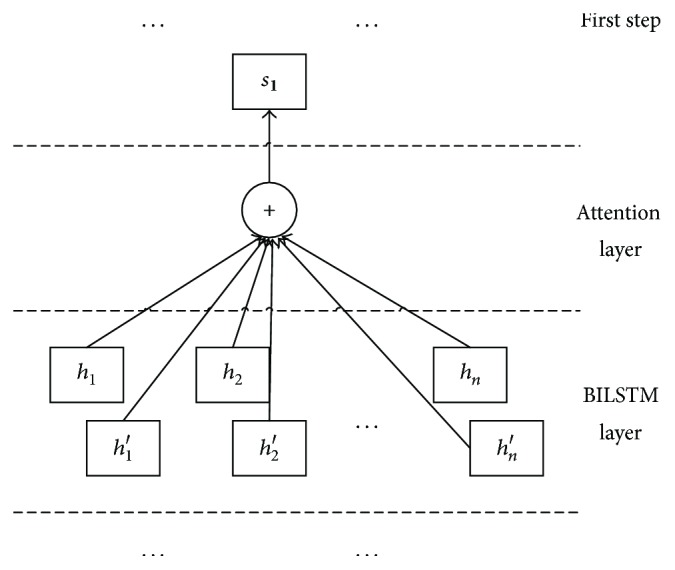
Attention layer.

After realizing preliminary entity extraction, we choose two entities as target entity pair in the sentence *W* = [*w*_1_, *w*_2_,…, *w*_*n*_]. The attention layer is depicted in Figure 3. Let *H* be a matrix consisting of the hidden state vectors [*h*_1_, *h*_1_′, *h*_2_, *h*_2_′,…, *h*_*n*_, *h*_*n*_′] in the BILSTM layer, and *H* is the input of the attention layer. Then attention based method represents the sentence that includes target entity pair as a weighted sum of these hidden state vectors.

(6) A=tanh⁡H,α=softmaxωTA,s1=tanh⁡HαT.

Here, *α* is the normalized weight vector and *ω* is a parameter vector. *s*_1_ is the initial state, in which we denote by *s*_1_ = Att(*X*; ***θ***_1_), and ***θ***_1_ represents all the parameters in this method.

After generating the initial state *s*_1_, the first decision will be made to judge if a sentence that includes target entity pair is a relation mention. We pass *s*_1_ to a softmax output layer to get *y*_*a*_, which is the probability of relation mention and nonrelation for a sentence. Finally, we can determine to take *a*_1_ or *a*_2_.

(7) $$\begin{array}{c} y_{a}=\text{softmax}\left(\;W_{sy}s_{1}+b_{y}\;\right). \end{array}$$

Here, *W* is weight matric and *b* is bias vector.

The objective function for *m* training instances is the negative log-likelihood:

(8) Jθ1=−12m∑i=1mt0ilog⁡yai0+t1ilog⁡yai1+λ2θ122

Here, *t*_0_^(*i*)^ and *t*_1_^(*i*)^ are the one-hot represented ground truth. *y*_*a*_^(*i*)^(0) and *y*_*a*_^(*i*)^(1) are the estimated probability for relation mention and nonrelation, respectively. *λ* is an *L*2 regularization hyperparameter.

To minimize *J*(***θ***_1_), we use a simple optimization technique called stochastic gradient descent (SGD).

### 3.4. Tree-LSTM

Unlike traditional sequence LSTM, Tree-LSTM [13] is constructed over a tree structure. As is known to all, the dependency tree is very useful for analyzing the relations between words. Two words may be far apart in the linear structure and separated by many unrelated words or preposition structure, but they are in hyponymy for the dependency tree. Therefore, we construct the Tree-LSTM over the dependency tree to represent relation mentions in a bottom-up way. Tree-LSTM can extract the core dependency relation between target entity pair and generate the transition state *s*_2_ in the decision process. The second decision on *s*_2_ performs preliminary relation extraction.

**Figure 4:**
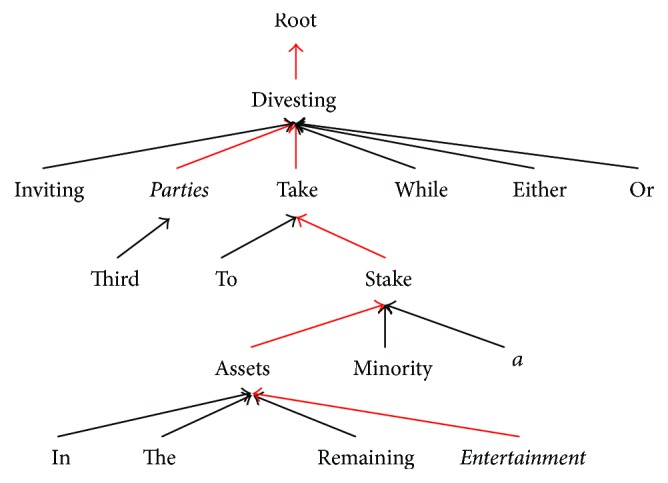
Dependency tree of a relation mention.

We take the relation mention “AFP_ENG_20030319.0879-R2” in Table 1 as an example to illustrate, and the two entity arguments are “third parties” and “Entertainment.” Firstly, we perform dependency parsing on the relation mention and generate the dependency tree, as shown in Figure 4. Instead of using the full mention boundary, we use head spans for entities directly. The entity head of “third parties” is “parties,” and the entity head of “Entertainment” is “Entertainment.” The core dependency relation between target entity pair is shown by red lines in Figure 4. So we use dependency tree as a backbone to construct Tree-LSTM. Moreover, for the convenience of implementation, we prune or pad dependency trees to keep the same depth and width.

Like BILSTM, each LSTM unit of Tree-LSTM takes continuous feature vector of a word as input. In addition to word embedding *e*_*t*_ and part-of-speech embedding *d*_*t*_, we use entity type embedding *t*_*t*_ and entity position embedding *l*_*t*_, to which entity type feature and entity position feature are mapped. We can get the entity type features from the preliminary results of entity extraction and get the entity position features by computing the relative distances of the current word to the two entity arguments. Unlike BILSTM, the LSTM unit does not accept hidden state vectors of the adjacent words and accept the hidden state vectors of all children nodes *h*_*tk*_ as input. The Tree-LSTM is developed from its leaf node in a recursive way up to the root, which is the common ancestor (“divesting” in Figure 4) of all the words. Then we carry out nonlinear transformation on the hidden state vector of the ancestor to generate *s*_2_, which is the final representation of relation mentions and serves as the transition state in the decision process. We denote *s*_2_ by *s*_2_ = Tree(*X*; ***θ***_2_), and ***θ***_2_ represents all the parameters in the Tree-LSTM.

After generating the transition state *s*_2_, the second decision will be made to classify the relation mention into a targeted type. Then *s*_2_ is passed to a softmax output layer to get *y*_*r*_, which is the probability of different types for a relation mention. Finally, we choose a type with the maximum probability, which determines to take *a*_3_ or *a*_4_….

(9) $$\begin{array}{c} y_{r}=\text{softmax}\left(\;W_{sy}s_{2}+b_{y}\;\right). \end{array}$$

Here, *W* is weight matric and *b* is bias vector. At each dependency tree, we use a softmax layer to predict the type for the root node given the inputs *X* observed at its children nodes.

The objective function for *m* training instances is the negative log-likelihood:

(10) $$\begin{array}{c} J\left(\;\theta_{2}\;\right)=-\frac{1}{m}\overset{m}{\underset{i=1}{\sum }}\log\left(\;y_{r}^{\left(i\right)}\;\right)+\frac{\lambda }{2}{\left\|\;\theta_{2}\;\right\|}_{2}^{2}. \end{array}$$

Here, *y*_*r*_^(*i*)^ is the estimated probability for the true type at each root node. The root node of Tree-LSTM is able to selectively incorporate information from each child. *λ* is an *L*2 regularization hyperparameter.

To minimize *J*(***θ***_2_), we use AdaGrad [14].

### 3.5. *Q*-Learning

*Q*-Learning algorithm [15] is a popular form of reinforcement learning and can be used to learn an optimal state-action value function *Q*(*s*, *a*) for the agent. The agent takes an action *a* in state *s* by consulting *Q*(*s*, *a*), which is a measure of the action's expected long-term reward. The aim is to maximize some cumulative rewards through a sequence of actions. As the state space is infinite in the decision process, it is impractical to obtain *Q*(*s*, *a*) for all possible state-action pairs.

For the above challenge, we approximate *Q*(*s*, *a*) using a neural network, which can represent *Q*(*s*, *a*) as a parameterized function *Q*_**η**_(*s*, *a*) = MLP(*ϕ*(*X*; ***θ***), *a*; **η**). *ϕ*(*X*; ***θ***) refers to *s*_1_ = Att(*X*; ***θ***_1_) and *s*_2_ = Tree(*X*; ***θ***_2_) above, where ***θ*** can be obtained by pretraining the deep learning models above and **η** represents the parameters in the neural network, which are learnt by performing stochastic gradient descent step with RMSprop [16].

To approximate the real value function *Q*^*π*^ as closely as possible, we measure the degree of approximation with the least squares error:

(11) $$\begin{array}{c} E_{\eta }=E\left[\;{\left(\;Q_{\pi }\left(\;s,a\;\right)-Q_{\eta }\left(\;s,a\;\right)\;\right)}^{2}\;\right]. \end{array}$$

In *Q*-Learning, we use the estimated value function *Q*_**η**_(*s*, *a*) instead of the real value function *Q*^*π*^(*s*, *a*). During each epoch, the updates of parameters aim to reduce the discrepancy between the estimation *Q*_**η**_(*s*, *a*) and the expectation *Q*^*π*^(*s*, *a*). The agent starts from a random *Q*_**η**_(*s*, *a*) and continuously updates its values by making the decisions and obtaining rewards. Then the agent can maximize its expected future rewards by choosing the action with the highest *Q*_**η**_(*s*, *a*′′). Finally, *Q*-Learning algorithm gets control policy *π* in the two-step decision process.  Algorithm 1 details the *Q*-Learning training procedure.

During the training procedure we pretrain BILSTM, the attention layer, and Tree-LSTM, respectively. The training parameters mainly include all the parameters ***θ***_0_ in BLSTM, all the parameters ***θ***_1_ in the attention layer, and all the parameters ***θ***_2_ in Tree-LSTM.

The functionality of the attention model in our RL method is very similar to that of a separate relation mention classification part in a pipeline. We use deep learning methods to represent words and sentences in the text and use RL to combine three tasks in the decision process, that are entity extraction, relation mention classification, and relation classification. The pipeline architecture just passes the information of entity extraction to relation extraction and does not enable information to flow in the global architecture. However, our RL method not only combines the above tasks sequentially but also globally makes decisions. At the beginning, the decisions have close to a random chance. After several epochs, they will be stabilizing. Meanwhile, the parameters in our architecture are globally updated and eventually converge. Therefore, our RL method can obtain feedback from decision-making and state changes and enable information to flow in the global architecture. The attention model connects entity extraction task with relation extraction task, thus helping us to realize the joint extraction of entities and relations. Experimental results demonstrate that our RL method performs slightly better than the pipeline method for both entity extraction and relation extraction, which shows that we are on the right track.

## 4. Experiments

### 4.1. Data

Most previous work has reported results on ACE2005 data set, so we evaluate our method on ACE2005 for joint extraction of entities and relations. We use three common metrics to evaluate the performance: microprecision (*P*), recall (*R*), and primary micro *F*1-scores (*F*1). An entity mention is correct when its entity type and the region of its head are correct, and a relation mention is correct when its relation type and both entity arguments are correct.

Data source for English in ACE2005 is as follows: 20% Newswire (NW), 20% Broadcast News (BN), 15% Broadcast Conversation (BC), 15% Weblog (WL), 15% Usenet Newsgroups/Discussion Forum (UN), and 15% Conversational Telephone Speech (CTS). The two small subsets UN and CTS are informal, so we remove them. In addition, in order to compare with state of the art, we employ the same method as previous work [17] to split and preprocess the data. Training set contains 351 documents, development set contains 80 documents, and testing set contains 80 documents.

### 4.2. Hyperparameters

We set up Python2.7 + Theano + Cuda7.5 environments to implement our method. We use the publicly available word embedding Glove [18] to initialize the word embedding table, and its dimension *n*_*e*_ is 300. We fix the dimension of part-of-speech embedding *n*_*d*_ and the dimension of entity type embedding *n*_*t*_ to 50 and fix the dimension of entity position embedding *n*_*l*_ to 5. Those feature embeddings are randomly initialized and allowed to be modified during training. In addition, we fix the state size of all the LSTM units to 200 and fix the dimensions of other hidden layers to 100. We use tanh for the nonlinear function.

We tune hyperparameters using development set to achieve high *F*1. The best hyperparameters are as follows. Dropout rate [19] is 0.5, minibatch size is 30, the constraint of max-norm regularization is equal to 3, and initial learning rate is 0.0005. The reward after each action is described in the Section 3.1. Therefore, for all the experiments below, we will directly employ the best hyperparameters.

### 4.3. Overall Performance

We run experiments to analyze the effectiveness of the various components of our joint extraction method.

Firstly, we compare the performance of BILSTM with a baseline system, LSTM for entity extraction task. We train models using training set and report models' performance on development set in Table 2. The result shows that BILSTM obtains better performance than LSTM on all evaluation metrics. Bidirectional model can actually improve the performance of sequence tagging task. Therefore, throughout the experiment, we will use BILSTM to extract entities.

Then, to demonstrate the effectiveness of the relation extraction component of our method, we carry out experiments on relation extraction when entities are known. We build a baseline system, CNN. In addition, we parse relation mentions using the Stanford neural dependency parser [20] and directly use Tree-LSTM extract relations. On the basis of Tree-LSTM, we use reinforcement learning method to control the process of relation extraction. We compare the performance of the above three methods on development set in Table 3. The result demonstrates that Tree-LSTM is better suited to extract relations than CNN, and reinforcement learning method obtains a substantial gain in recall-score over Tree-LSTM with 3.7%. Therefore, in the rest of the experiment, we will use reinforcement learning method based on Tree-LSTM to extract relations.

Finally, we demonstrate the effectiveness of our joint extraction method. We build a pipelined system, which directly connects the entity extraction component and the relation extraction component above. To be specific, the pipelined system first trains the entity extraction model and then builds a separate relation extraction model using the detected entities. Our joint system is based on the pipelined system. The joint system uses attention based method to pass entity information to relation extraction and updates the parameters in all the components simultaneously during the training procedure for *Q*-Learning, which realizes the joint extraction of entities and relations. We compare the performance of the two systems on development set in Table 4. The result demonstrates that our joint system slightly improves the performance of entity extraction and significantly improves the performance of relation extraction. Therefore, the experiments show that our method is effective and practical.

**Figure 5:**
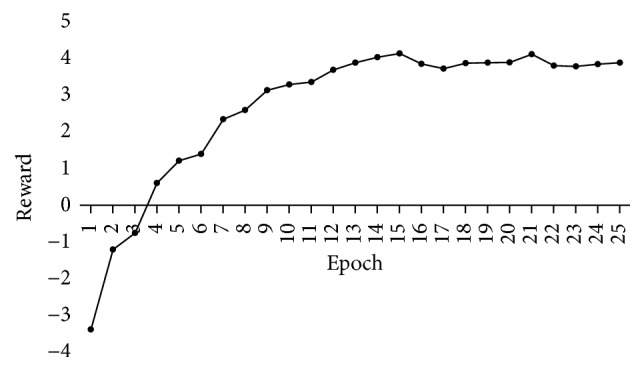
Learning curve of average reward.

**Figure 6:**
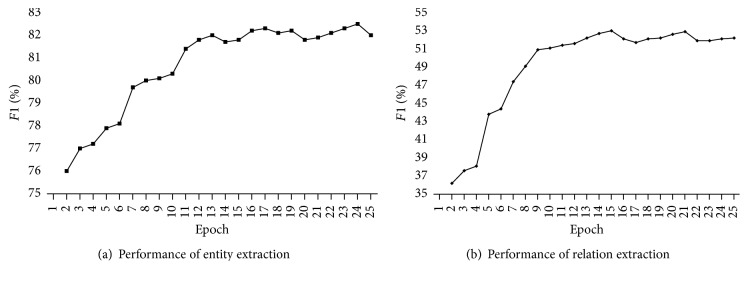
Learning curves of the performance.

We will clearly show the process of the above experiments.  Figure 5 shows the average reward after each training epoch. At the beginning of training, the reward is negative, because the agent takes actions randomly. But with the increase of epoch number, the reward improves gradually.  Figure 6 shows the learning curves of the performance for entity extraction and relation extraction. The *F*1-score in both (a) and (b) increases simultaneously. From the two figures, we can clearly see that all the metrics significantly improve and then stabilize after 13 epochs of training. So we set the number of training epochs as 13.

### 4.4. Comparison with State of the Art

Now deep learning methods achieve state-of-the-art performance in end-to-end relation extraction task. Miwa and Bansal [21] stacked bidirectional tree-structured LSTM-RNNs on bidirectional sequential LSTM-RNNs to extract entities and relations between them, which could capture both word sequence and dependency tree substructure information. The method is denoted by SPTree.  Table 5 compares our joint extraction method with SPTree on the testing set and shows that our method performs slightly better than SPTree for both entity mentions and relation mentions. Although our method is not comparable with SPTree in precision-score, our method outperforms the best results of SPTree in recall-score. The main reason is that the reward after each action in reinforcement learning may play an important role.

### 4.5. Analysis

**Figure 7:**
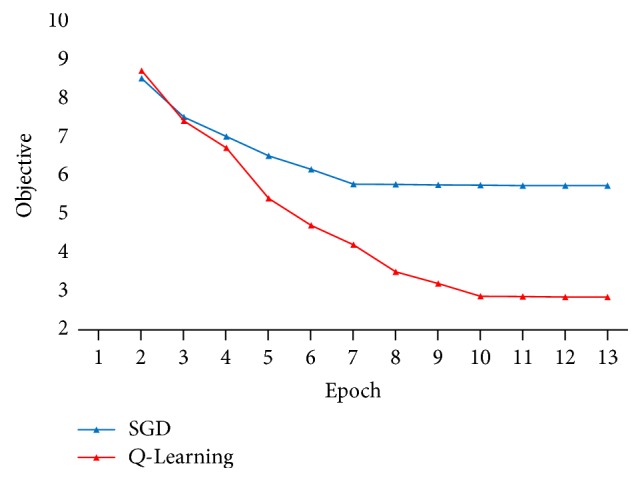
Objective values in the attention model.

**Figure 8:**
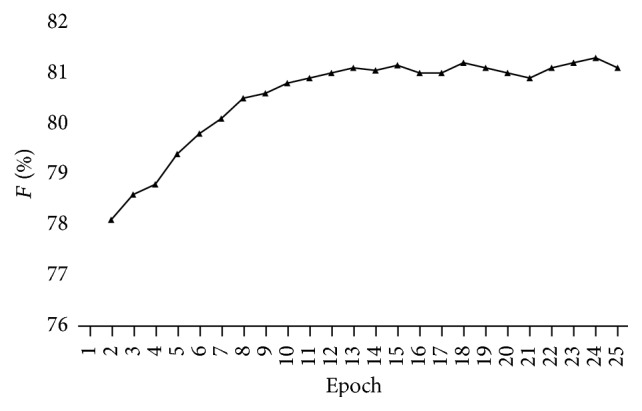
Performance of relation mention classification.

We pretrain the attention model which is used for relation mention classification. Relation mention classification is always processed in a very unbalanced corpus, where most sentences are not a relation mention. From Figure 7, we see that the SGD algorithm gets to the minimum objective fast, but the objective function's value is a bit high. That means that during the pretraining of the attention model there would be a huge loss. The parameters in the attention layer are updated to accepted values, which are prepared for *Q*-Learning. When we do *Q*-Learning, we learn a stacked MLP on top of the attention model (without softmax output layer). From Figure 7, we see that *Q*-Learning takes more epochs to converge but reduces the value of the objective function in the first stage of the MDP. That means that our reinforcement learning method is effective despite the huge loss and poor initialization in the pretraining of the attention model. Moreover, Figure 8 shows the learning curves of the performance for relation mention classification. We can see that our reinforcement learning method gets good performance in the *F*1-score, which is also a proof of our effectiveness.

## 5. Related Work

As for joint extraction of entities and relations, the research has been dominated by four methods. The first one is structured prediction. Li and Ji [17] presented an incremental joint framework to simultaneously extract entity mentions and relations using structured perceptron with efficient beam-search. The second one is integer linear programming. Dan and Yih [22] studied global inference for entity and relation identification via a linear programming formulation. The third one is card-pyramid parsing. Kate and Mooney [23] presented a new method for joint entity and relation extraction using card-pyramid parsing. The last one is global probabilistic graphical models. Yu and Lam [24] jointly identified entities and extracted relations in encyclopedia text via a graphical model approach.

Recently, deep learning methods have been widely used in many research areas with the aim of reducing the number of handcrafted features. However, the only work of end-to-end (joint) extraction of relations between entities with deep learning methods is due to Miwa and Bansal [21], and most researchers simply solve entity extraction, relation classification, or relation extraction separately. Chiu and Nichols [25] presented a novel neural network architecture for named entity recognition, which automatically detected word- and character-level features using a hybrid bidirectional LSTM and CNN architecture. Zhang et al. [26] proposed bidirectional long short-term memory networks (BLSTM) to model the sentence with complete, sequential information about all words for relation classification. Nguyen and Grishman [27] departed from these traditional approaches with complicated feature engineering by introducing a convolutional neural network for relation extraction.

At present, the research of reinforcement learning has risen. El-Laithy and Bogdan [28] presented a reinforcement learning framework for spiking networks with dynamic synapses. Mousavi et al. [29] discussed the notion of context transfer in reinforcement learning tasks. However, few researchers apply reinforcement learning in text processing tasks. We use both reinforcement learning and deep learning to simultaneously extract entities and relations from unstructured texts. To the best of our knowledge, there has been no work on employing reinforcement learning for information extraction so far. This paper is the first attempt to fill in that gap and provides a good thinking way for future research in this area.

## 6. Conclusions

In this paper we define and research the joint extraction of entities and relations. We model the task as a two-step decision process in reinforcement learning. In addition, we use deep learning methods to represent the state in the decision process. Attention based method can pass entity information to relation extraction task. During the training procedure for *Q*-Learning, all the parameters are updated simultaneously to realize the interaction and feedback of entity information and relation information. The reward after each action in reinforcement learning apparently helps to improve the recall-score. Under the same experimental conditions, our method outperforms the state-of-the-art method in *F*1-score of entity mentions and relation mentions. In future work, we plan to perfect the model of the two-step decision process and optimize the *Q*-Learning algorithm.

## Figures and Tables

**Algorithm 1:**
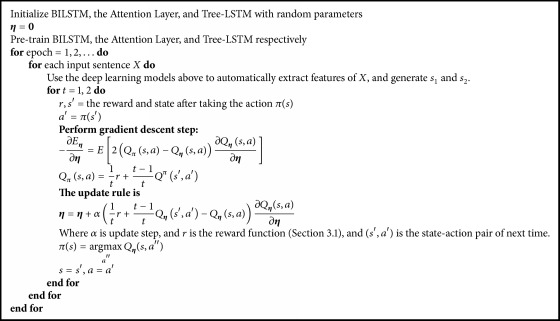
Training procedure for *Q*-Learning.

**Table 1:** A sentence in ACE2005 dataset.

Sentence	While either divesting or inviting third parties to take a minority stake in the remaining Entertainment assets.	
Entity ID = “AFP_ENG_20030319.0879-E24”	Type = “ORG” Subtype = “Commercial”	third parties

Entity ID = “AFP_ENG_20030319.0879-E25”	Type = “ORG” Subtype = “Entertainment”	Entertainment

Relation ID = “AFP_ENG_20030319.0879-R2”	Type = “ORG-AFF” Subtype = “Investor-Shareholder”	RefID = “AFP_ENG_20030319.0879-E24” Role = “Arg-1”
		RefID = “AFP_ENG_20030319.0879-E25” Role = “Arg-2”

**Table 2:** Performance for entity extraction task.

Method	Entity		
Score	*P* (%)	*R* (%)	*F*1 (%)
LSTM	81.0	78.1	79.5
BILSTM	82.5	79.8	81.1

**Table 3:** Performance for relation extraction task.

Method	Relation		
Score	*P* (%)	*R* (%)	*F*1 (%)
CNN	63.1	52.9	57.6
Tree-LSTM	63.9	54.1	58.6
RL	63.6	59.4	61.4

**Table 4:** Performance of two extraction systems.

Method	Entity			Relation		
Score	*P* (%)	*R* (%)	*F*1 (%)	*P* (%)	*R* (%)	*F*1 (%)
Pipeline	82.5	79.8	81.1	60.2	43.9	50.8
Joint	83.6	80.4	82.0	60.6	45.9	52.2

**Table 5:** Comparison with state of the art.

Method	Entity			Relation		
Score	*P* (%)	*R* (%)	*F*1 (%)	*P* (%)	*R* (%)	*F*1 (%)
SPTree	85.5	81.2	83.3	65.8	42.9	51.9
Joint	85.0	**82.4**	83.7	65.9	**45.3**	53.7
